# Diabetes-Related Distress, Depression and Distress-Depression among Adults with Type 2 Diabetes Mellitus in Malaysia

**DOI:** 10.1371/journal.pone.0152095

**Published:** 2016-03-22

**Authors:** Boon-How Chew, Rimke Vos, Sherina Mohd-Sidik, Guy E. H. M. Rutten

**Affiliations:** 1 Department of Family Medicine, Faculty of Medicine and Health Sciences, Universiti Putra Malaysia, 43400, Serdang, Selangor, Malaysia; 2 Julius Center for Health Sciences and Primary Care, University Medical Center Utrecht, Department of General Practice, Huispost Str.6.131, P.O. Box 85500, 3508 GA, Utrecht, The Netherlands; 3 Department of Psychiatry, Faculty of Medicine and Health Sciences, Universiti Putra Malaysia, 43400, Serdang, Selangor, Malaysia; Chiba University Center for Forensic Mental Health, JAPAN

## Abstract

Type 2 diabetes mellitus (T2DM) brings about an increasing psychosocial problem in adult patients. Prevalence data on and associated factors of diabetes related distress (DRD) and depression have been lacking in Asia. This study aimed to examine the prevalence of DRD and depression, and their associated factors in Asian adult T2DM patients. This study was conducted in three public health clinics measuring DRD (Diabetes Distress Scale, DDS), and depression (Patient Health Questionnaire, PHQ). Patients who were at least 30 years of age, had T2DM for more than one year, with regular follow-up and recent laboratory results (< 3 months) were consecutively recruited. Associations between DRD, depression and the combination DRD-depression with demographic and clinical characteristics were analysed using generalized linear models. From 752 invited people, 700 participated (mean age 56.9 years, 52.8% female, 52.9% Malay, 79.1% married). Prevalence of DRD and depression were 49.2% and 41.7%, respectively. Distress and depression were correlated, spearman’s r = 0.50. Patients with higher DRD were younger (OR 0.995, 95% CI 0.996 to 0.991), Chinese (OR 1.2, 95% CI 1.04 to 1.29), attending Dengkil health clinic (OR 1.1, 95% CI 1.00 to 1.22) and had higher scores on the PHQ (OR 1.1, 95% CI 1.04 to 1.06). Depression was less likely in the unmarried compared to divorced/separately living and those attending Dengkil health clinic, but more likely in patients with microvascular complications (OR 1.4, 95% CI 1.06 to 1.73) and higher DDS (OR 1.03, 95% CI 1.02 to 1.03). For the combination of DRD and depression, unemployment (OR 4.7, 95% CI 1.02 to 21.20) had positive association, whereas those under medical care at the Salak health clinics (OR 0.28, 95% CI 0.12 to 0.63), and those with a blood pressure > 130/80 mmHg (OR 0.53, 95% CI 0.32 to 0.89) were less likely to experience both DRD and depression. DRD and depression were common and correlated in Asian adults with T2DM at primary care level. Socio-demographic more than clinical characteristics were related to DRD and depression.

## Background

Type 2 diabetes mellitus (T2DM) brings about an increasing psychosocial problem in adult patients [1–5]. Life routines and social functioning [6, 7] alter by the recurrent symptoms, regular medication taking and higher demands for healthy life-styles [8–10]. Coping with threats on physical health [11, 12], communication with healthcare professionals about personal health beliefs and illness perceptions [13] may be burdensome.

The most common psychological disorders experienced by adult T2DM patients are diabetes-related distress (DRD) and depressive disorders [14–17]. A study in the United States (US) showed that over 18 months, T2DM patients who ever experienced major depressive disorder (MDD) and DRD were about 20% and 30%, respectively [16]. Also in the United Kingdom, adults with T2DM were, after controlling for age, gender, ethnicity, and socioeconomic status, more likely to suffer from these common mental disorders [14]. Reported prevalence of DRD in Europe and the US is between 15% and 20% [18–20]. However, prevalence rate from Asian countries are lacking.

DRD and depression in adult T2DM patients have been associated with somatic symptoms [21], smoking behaviours [22] and disease control [23, 24]. A larger study with longer follow-up demonstrated an association between decreased quality of life with either DRD or depression [25]. Also, diabetes-related complications and mortality are associated with DRD and depression [26, 27]. Other risk factors for DRD and/or depression in T2DM patients include adherence to the complex therapeutic requirement [28], insulin initiation [29]; the quality of social support [30] and inter-personal relationships with others including spouses [31, 32].

However, risk factors for general stress, DRD and depression are not similar across different geographical regions and cultural backgrounds in terms of socio-demographic-clinical characteristics. For example in Australia and Malaysia, female gender [15, 33] was associated with general stress; in US, young age, low education levels, low household income, obesity, current smoking, no leisure-time physical activity and presence of one or more micro- or macro-vascular complications were associated with distress [34]. In China [35], DRD and older age were associated with depression. In a Malaysian study a family history of psychiatric illnesses, current alcohol drinking, and Indian ethnicity were associated with depression and stress [15]. The present study aimed to determine the prevalence of DRD, depression and DRD-depression combined in Asian patients with T2DM. In addition, the association between socio-demographic-clinical characteristics and DRD, depression and the combination will be explored. These two affective disorders were not clearly distinguished until a recent review by Fisher et al. [36]. DRD relates to diabetes-related concerns about disease management, (social) support, emotional burden, and access to diabetes care [37]; while depression is not confined to the diabetes context and its measurement provides an opportunity in assessing the severity of depressive symptoms in a broader spectrum of emotional distress [36].

## Materials and Methods

### Ethics statement

This research project was approved by the Medical Research Ethics Committee (MREC), Ministry of Health, Malaysia. It conformed to the provisions of the Declaration of Helsinki in 1995 (as revised in Edinburgh 2000). All investigations on human subjects had obtained their written informed consent and patient anonymity was preserved throughout the study.

### Setting

Participants were recruited in 2012–2013 from three public health clinics in Seri Kembangan (~10 general practitioners), Dengkil (~5 general practitioners) and Salak (~10 general practitioners) in Malaysia. The first one is an urban clinic at the vicinity of the Chinese communities and visited mainly by Chinese patients. Dengkil health clinic is located in a rural area and mainly visited by the Malay and the Indian population. Salak health clinic is another rural clinic, which is mainly visited by the Malay. The variety of the sites provided a broad range of Asian T2DM patients.

### Participants

Eligible patients were at least 30 years of age, known with T2DM for more than one year, with regular follow-up of at least three visits in the past year and with recent laboratory results (< 3 months). T2DM was assumed to be present if there was a documented diagnosis of diabetes mellitus according to the 1999 World Health Organization criteria [38] or/and (ii) people were treated with lifestyle modification, oral anti-hyperglycaemic agents or insulin. Patients were excluded if they were pregnant or lactating, having psychiatric/psychological disorders that could impair judgments and memory, or patients who could not read or understand English, Malay or Mandarin. The psychiatric/psychological disorder as an exclusion criterion was specified mainly for psychoses (schizophrenia and bipolar disorders) and dementia. It did not exclude those with mood disorders except those with psychoses and dementia. However, throughout the study there was no exclusion of adults with T2DM because of affective disorders but a few were due to psychoses. Included patients gave written informed consent and received the questionnaires according to the language they preferred. Trained research assistants interviewed the patients who for some reasons were not able to self-administer the questionnaires.

### Diabetes related distress

Diabetes related distress (DRD) was measured by using the 17-item Diabetes Distress Scale (DDS) [37, 39]. This instrument assesses problems and difficulties related to diabetes during the past month on a Likert scale from 1 (not a problem) to 6 (a very serious problem) [37]. The DDS provides a total DRD scale score and 4 subscale scores, addressing emotional burden (EB), physician-related distress (PD), regimen-related distress (RD), and diabetes-related interpersonal distress (ID). An overall mean score of less than 2.0 indicates little to no distress, a score between 2.0 and 2.9 indicates moderate distress and a score of 3.0 or higher is defined as a high level of distress [40]. A local translation and validation study of DDS Malay version showed high internal consistency (Cronbach’s α = 0.94), the 1-month test–retest reliability value was Spearman’s correlation *r* = 0.33 (P = 0.009) [41].

### Depression

Depression was measured by the 9-item Patient Health Questionnaire (PHQ) [42, 43], which refers to symptoms experienced during the last two weeks. The PHQ includes nine items, each score ranges from 0 (not at all) to 3 (nearly every day), with a total score ranging from 0–27. PHQ scores with cut-off point 5, 10, 15, and 20 represent mild, moderate, moderately severe, and severe depression, respectively [42]. A validated Malay version of the PHQ was used, with sufficient internal consistency (Cronbach’s α = 0.70) [44].

### Demographic and clinical variables

Demographic data on age, gender, ethnicity, religion, marital status, educational level, employment status, exercise and smoking status were collected by using self-report questionnaires. Data on hypertension (SBP ≥ 130 mm Hg or DBP ≥ 80 mm Hg or patients using antihypertensive medication) and dyslipidaemia ((LDL-cholesterol > 2.6 mmol/L or triglyceride (TG) > 1.7 mmol/L or HDL-cholesterol < 1.1 mmol/L, or the use of lipid-lowering medication), diabetes-related complications (retinopathy, micro-albuminuria, proteinuria, serum creatinine > 150 μmol/L or estimated glomerular filtration rate < 60ml/min), diabetic foot problems (foot deformity, current ulcer, amputation, peripheral neuropathy or peripheral vascular disease), ischemic heart disease and cerebrovascular disease, duration of diabetes, HbA1clevel, blood pressure, lipids, and the number and type of medication use were retrieved from the patient’s medical record by using case record forms. Micro-albuminuria, proteinuria, serum creatinine > 150 μmol/L or estimated glomerular filtration rate < 60ml/min indicated presence of nephropathy. Retinopathy, nephropathy and diabetic foot problems were grouped as microvascular complications. Ischemic heart disease and cerebrovascular disease or stroke were classified as macrovascular complications.

### Statistical Analyses

A sample size of 494 patients was based on an estimated prevalence of DRD at 25% with a power of 0.80 and an alpha of 5%. Taking into consideration about 30% of incomplete/missing data in the patient’s medical record and questionnaires, the requested sample size was 642.

Data analyses were performed with SPSS version 22.0 (IBM, Chicago, IL). Chi-square tests were used to analyse the difference in prevalence between DRD, depression, and the combination of both across different categories of the socio-demographic and clinical variables. To analyse the association of the demographic and clinical variables with DRD, its subscales and depression, Generalized Linear Models (GLM) were used with either DRD or depression or the combination DRD-depression as outcome variable and adjusted for age, gender and ethnicity. Since DRD and depression were skewed to the right, GLM with gamma distribution and log link function was used to analyse DDS and PHQ scores in the interval form. The gamma distribution is a reasonable choice because its flexible distribution accommodates well for positive and continuous variables and it incorporates the assumption that the standard deviation is proportional to the mean. Those demographic and clinical factors with a *p* value < 0.20 from the univariable analyses were included in the final multivariable GLM analyses. To analyse the associations with the combination of DRD and depression, GLM for binomial distribution with logit link function was used. This combined variable included participants with a mean total DDS score ≥ 2 (any degree of DRD from moderate to high level DRD) and a mean total PHQ score ≥ 5 (any degree of depression from mild to severe depression). Therefore, the reference category was DDS < 2 and PHQ < 5. The same confounders’ selection rules were applied as described above. In all the models, Q-Q plots gave indication of normality, the residual plots indicated fulfilment of linearity and homogeneity assumptions and model fitting encountered no problems.

## Results

The response rate was 93.1% (700/752). Participants’ mean age was 56.9 years, and their median (interquartile range) for diabetes duration was 4.0 (6.00) years. More than half of the participants were female, the majority was Malay (52.8%), married or living with partners (79.1%), and most did not have a tertiary education (89.1%) (Table 1). About 80% of the patients had hypertension and anti-hypertensive usage was almost 90% (Table 2).

**Table 1:** Socio-demographic characteristic according to the DDS and PHQ categories. N = 697 (DDS) and 684 (PHQ).

Socio-demography		Total, n	Mean total DDS score, n (%)			Total, n	PHQ Depression severity in 3 categories, n (%)			
			< 3	≥ 3	*t/X*^2^		None	Mild	Moderate-Severe	*F/X*^2^
Health Clinic	Seri Kembangan	223	159 (71.3)	64 (28.7)	36.69**	218	113 (51.8)	74 (33.9)	31 (14.2)	6.61
	Dengkil	122	87 (71.3)	35 (28.7)		121	72 (59.5)	36 (29.8)	13 (10.7)	
	Salak	352	315 (89.5)	37 (10.5)		345	214 (62.0)	99 (28.7)	32 (9.3)	
Age, year	Mean (SD)	661	57.3 (10.06)	55.1 (9.74)	2.20*	682	57.5 (9.45)	55.9 (11.21)	55.7 (10.16)	2.25
	≤ 50	185	143 (77.3)	42 (22.7)	5.44	183	90 (49.2)	70 (38.3)	23 (12.6)	10.38*
	51–60	271	213 (78.6)	58 (21.4)		265	164 (61.9)	69 (26.0)	32 (12.1)	
	> 60	239	204 (85.4)	35 (14.6)		234	143 (61.1)	70 (29.9)	21 (9.0)	
Diabetes Duration, year	Median (IQR)	641	4.0 (6.00)	4.0 (6.00)	31.50^†^	641	4.0 (5.00)	5.0 (6.00)	5.0 (7.00)	1.12^‡^
	< 5	339	271 (79.9)	68 (20.1)	0.05	333	203 (61.0)	93 (27.9)	37 (11.1)	3.59
	5–9.9	190	153 (80.5)	37 (19.5)		188	107 (56.9)	62 (33.0)	19 (10.1)	
	≥ 10	145	117 (80.7)	28 (19.3)		140	74 (52.9)	47 (33.6)	19 (13.6)	
Gender	Female	367	292 (79.6)	75 (20.4)	0.35	364	206 (56.6)	113 (31.0)	45 (12.4)	1.82
	Male	327	266 (81.3)	61 (18.7)		317	192 (60.6)	95 (30.0)	30 (9.5)	
Ethnicity	Malay	365	311 (85.2)	54 (14.8)	12.62*	361	218 (60.4)	98 (27.1)	45 (12.5)	7.57
	Chinese	162	117 (72.2)	45 (27.8)		157	89 (56.7)	49 (31.2)	19 (12.1)	
	Indian	164	129 (78.7)	35 (21.3)		161	88 (54.7)	61 (37.9)	12 (7.5)	
Religion	No religion	33	21 (63.6)	12 (36.4)	22.71**	30	14 (46.7)	12 (40.0)	4 (13.3)	16.03
	Moslem	373	320 (85.8)	53 (14.2)		369	225 (61.0)	99 (26.8)	45 (12.2)	
	Buddhist	82	57 (69.5)	25 (30.5)		80	44 (55.0)	23 (28.8)	13 (16.3)	
	Hinduism/Sikh	148	115 (77.7)	33 (22.3)		145	78 (53.8)	57 (39.3)	10 (6.9)	
	Christian/Catholic	22	15 (68.2)	7 (31.8)		21	13 (61.9)	5 (23.8)	3 (14.3)	
	Others	37	32 (86.5)	5 (13.5)		37	24 (64.9)	12 (32.4)	1 (2.7)	
Religiosity	Religious	592	488 (82.4)	104 (17.6)	10.07*	583	348 (59.7)	169 (29.0)	66 (11.3)	6.12
	Unsure	25	16 (64.0)	9 (36.0)		25	12 (48.0)	10 (40.0)	3 (12.0)	
	Not religious	76	54 (71.1)	22 (28.9)		72	36 (50.0)	30 (41.7)	6 (8.3)	
Marital status	Married/living with a partner	548	443 (80.8)	105 (19.2)	2.35	541	322 (59.5)	161 (29.8)	58 (10.7)	6.86
	Widow	98	81 (82.7)	17 (17.3)		97	54 (55.7)	33 (34.0)	10 (10.3)	
	Divorced/separated	21	15 (71.4)	6 (28.6)		18	7 (38.9)	6 (33.3)	5 (27.8)	
	Single	26	19 (73.1)	7 (26.9)		24	15 (62.5)	7 (29.2)	2 (8.3)	
Education	Never visited school	45	36 (80.0)	9 (20.0)	0.26	45	25 (55.6)	15 (33.3)	5 (11.1)	1.42
	Primary & secondary	566	454 (80.2)	112 (19.8)		553	328 (59.3)	166 (30.0)	59 (10.7)	
	Tertiary	75	62 (82.7)	13 (17.3)		75	41 (54.7)	23 (30.7)	11 (14.7)	
Employment	Retired/home manager	370	305 (82.4)	65 (17.6)	5.93	361	212 (58.7)	109 (30.2)	40 (11.1)	13.29*
	Employed	313	248 (79.2)	65 (20.8)		309	185 (59.9)	92 (29.8)	32 (10.4)	
	Unemployed	11	6 (54.5)	5 (45.5)		11	1 (9.1)	6 (54.5)	4 (36.4)	
Exercise	No	295	240 (81.4)	55 (18.6%)	0.73	292	167 (57.2)	91 (31.2)	34 (11.6)	6.29
	≤ 3 times in a week	230	181 (78.7)	49 (21.3)		222	120 (54.1)	78 (35.1)	24 (10.8)	
	> 3 times in a week	168	137 (81.5)	31 (18.5)		166	109 (65.7)	40 (24.1)	17 (10.2)	
Smoking	Never	529	425 (80.3)	104 (19.7)	0.07	521	305 (58.5)	159 (30.5)	57 (10.9)	0.48
	Stop > 5 years	60	49 (81.7)	11 (18.3)		58	33 (56.9)	17 (29.3)	8 (13.8)	
	Stop ≤ 5 years and active smoker	105	84 (80.0)	21 (20.0)		102	59 (57.8)	32 (31.4)	11 (10.8)	

* Chi-square *P* value < 0.05

** Chi-square *P* value < 0.0001

^†^ Mann-Whitney U test, P value = 0.42

^‡^ Kruskal-Wallis test, P value = 0.57

*t* = independent t-test, *X*^2^ = Chi-square test. *F* = one-way analysis of variance (ANOVA). DDS = Diabetes Distress Scale 17 items, IQR = interquartile range, PHQ = Patient Health Questionnaire 9 items, PHQ depression severity: 0 (none = 0–4), 1 (mild = 5–9), 2 (moderate = 10–14), 3 moderately severe = 15–19) & 4 (severe = 20–27), SD = standard deviation

**Table 2:** Clinical parameters according to the DDS and PHQ categories.

Clinical parameters		Mean total DDS score, n (row %)				PHQ Depression severity in 3 categories, n (%)				
		Total, n	< 3	≥ 3	*t/X*^2^	Total, n	None	Mild	Moderate-Severe	*F/X*^2^
HbA1c, %	Mean (SD)	592	8.5 (2.15)	8.4 (2.02)	0.67	608	8.4 (2.05)	8.6 (2.22)	8.8 (2.42)	1.51
Hypertension	No	149	119 (79.9)	30 (20.1)	0.07	146	85 (58.2)	46 (31.5)	15 (10.3)	0.22
	Yes	537	434 (80.8)	103 (19.2)		527	306 (58.1)	160 (30.4)	61 (11.6)	
BP, mmHg	SBP, man (SD)	658	138.0 (17.31)	131.9 (17.7)	3.61**	679	137.8 (17.52)	136.8 (17.78)	132.7 (17.67)	2.64
	DBP, mean (SD)	658	79.6 (12.17)	77.1 (11.05)	2.11*	679	79.6 (12.09)	78.9 (12.08)	78.2 (12.90)	0.54
	BP > 130/80	480	399 (83.1)	81 (16.9)	6.92*	473	281 (59.4)	142 (30.0)	50 (10.6)	0.97
	BP ≤130/80	212	158 (74.5)	54 (25.5)		206	115 (55.8)	65 (31.6)	26 (12.6)	
Dyslipidaemia	No	407	352 (86.5)	55 (13.5)	19.99**	400	242 (60.5)	119 (29.8)	39 (9.8)	3.88
	Yes	263	191 (72.6)	72 (27.4)		258	138 (53.5)	85 (32.9)	35 (13.6)	
LDL-C, mmol/L	Mean (SD)	536	2.9 (0.98)	3.0 (0.99)	-0.68	554	2.9 (0.94)	3.0 (1.05)	3.2 (1.15)	2.40
	LDL-C > 2.6	334	256 (76.6)	78 (23.4)	3.79	329	187 (56.8)	99 (30.1)	43 (13.1)	1.76
	LDL-C ≤ 2.6	229	191 (83.4)	38 (16.6)		225	138 (61.3)	65 (28.9)	22 (9.8)	
HDL-C, mmol/L	Mean (SD)	539	1.0 (0.31)	1.0 (0.39)	-2.30*	557	1.0 (0.29)	1.0 (0.30)	1.1 (0.45)	3.16*
	HDL-C < 1.1	407	327 (80.3)	80 (19.7)	0.91	402	237 (59.0)	122 (30.3)	43 (10.7)	1.44
	HDL-C ≥ 1.1	159	122 (76.7)	37 (23.3)		155	90 (58.1)	43 (27.7)	22 (14.2)	
TG, mmol/L	Mean (SD)	539	2.0 (1.38)	1.8 (1.01)	1.40	557	1.9 (1.30)	2.0 (1.11)	2.0 (1.68)	0.10
	TG > 1.7	250	203 (81.2)	47 (18.8)	0.96	249	135 (54.2)	88 (35.3)	26 (10.4)	7.10*
	TG ≤1.7	316	246 (77.8)	70 (22.2)		308	192 (62.3)	77 (25.0)	39 (12.7)	
Total-C, mmol/L	Mean (SD)	588	4.8 (1.17)	4.9 (1.07)	-0.38	604	4.7 (1.08)	4.9 (1.21)	5.1 (1.40)	4.28*
	Total-C > 4.5	357	278 (77.9)	79 (22.1)	2.11	349	197 (56.4)	107 (30.7)	45 (12.9)	1.52
	Total-C ≤ 4.5	259	214 (82.6)	45 (17.4)		255	155 (60.8)	74 (29.0)	26 (10.2)	
Any diabetes complication	No	610	496 (81.3)	114 (18.7)	1.58	600	356 (59.3)	178 (29.7)	66 (11.0)	2.43
	Yes	86	65 (75.6)	21 (24.4)		83	42 (50.6)	31 (37.3)	10 (12.0)	
Any microvascular complication	No	643	523 (81.3)	120 (18.7)	2.91	632	374 (59.2)	190 (30.1)	68 (10.8)	3.02
	Yes	53	38 (71.7)	15 (28.3)		51	24 (47.1)	19 (37.3)	8 (15.7)	
Any macrovascular complication	No	650	523 (80.5)	127 (19.5)	0.01	638	375 (58.8)	191 (29.9)	72 (11.3)	2.41
	Yes	42	34 (81.0)	8 (19.0)		41	20 (48.8)	17 (41.5)	4 (9.8)	
OHA	No	61	48 (78.7)	13 (21.3)	0.14	59	30 (50.8)	19 (32.2)	10 (16.9)	2.56
	Yes	631	509 (80.7)	122 (19.3)		620	365 (58.9)	189 (30.5)	66 (10.6)	
Insulin	No	420	338 (80.5)	82 (19.5)	0.31	417	255 (61.2)	122 (29.3)	40 (9.6)	5.55
	1 type	191	152 (79.6)	39 (20.4)		184	98 (53.3)	59 (32.1)	27 (14.7)	
	≥ 2 types	80	66 (82.5)	14 (17.5)		77	41 (53.2)	27 (35.1)	9 (11.7)	
Number of AHA agents	No	82	61 (74.4)	21 (25.6)	8.38*	82	46 (56.1)	29 (35.4)	7 (8.5)	13.73*
	1 type	205	165 (80.5)	40 (19.5)		202	111 (55.0)	68 (33.7)	23 (11.4)	
	2 types	203	156 (76.8)	47 (23.2)		196	105 (53.6)	70 (35.7)	21 (10.7)	
	≥ 3 types	201	174 (86.6)	27 (13.4)		198	132 (66.7)	41 (20.7)	25 (12.6)	
LLA	No	156	130 (83.3)	26 (16.7)	1.06	151	79 (52.3)	57 (37.7)	15 (9.9)	4.57
	Yes	535	426 (79.6)	109 (20.4)		527	315 (59.8)	151 (28.7)	61 (11.6)	
APA	No	614	495 (80.6)	119 (19.4)	0.16	605	354 (58.5)	183 (30.2)	68 (11.2)	0.40
	Yes	75	59 (78.7)	16 (21.3)		71	39 (54.9)	24 (33.8)	8 (11.3)	

* Chi-square *P* value < 0.05

** Chi-square *P* value < 0.0001

*t* = independent t-test, *X*^2^ = Chi-square test. *F* = one-way analysis of variance (ANOVA). DDS = Diabetes Distress Scale 17 items, PHQ = Patient Health Questionnaire 9 items, PHQ depression severity: 0 (none = 0–4), 1 (mild = 5–9), 2 (moderate = 10–14), 3 (moderately severe = 15–19) & 4 (severe = 20–27). BP = blood pressure, DBP = diastolic blood pressure, LDL-C = low-density lipoprotein cholesterol, HDL-C = high-density lipoprotein cholesterol, SBP = systolic blood pressure, SD = standard deviation, TG = triglycerides, Total-C = total cholesterol. OHA = Oral Hypoglycaemic Agent, AHA = anti-hypertensive agent, LLA = lipid-lowering agent, APA = anti-platelet agent

### Prevalence of DRD and depression, related to different socio-demographic and clinical variables

**Fig 1:**
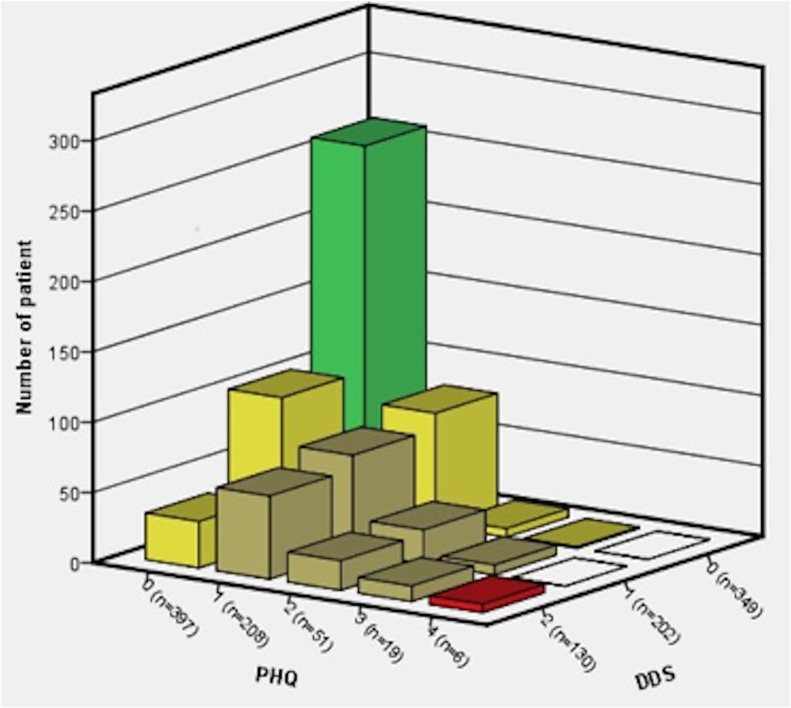
Distribution of depression within diabetes-related distress categories, n = 684. PHQ = Patient Health Questionnaire 9 items, depression severity: 0 (none = 0–4), 1 (mild = 5–9), 2 (moderate = 10–14), 3 (moderately severe = 15–19) & 4 (severe = 20–27). DDS = Diabetes Distress Scale 17 items, categories: 0 (no distress), 1(moderate distress) and 2 (high distress worthy of clinical attention)

The median (interquartile range) scores for the DDS and PHQ were 33.0 (23.00) and 4.0 (6.00), respectively. The prevalence of DRD was 49.2%, and moderate DRD was most frequently reported (29.7%). The prevalence of any degree of depression was 41.7%, with mild depressive symptoms most often present (30.6%). The combination of both DRD and depression was found in 14.0% of the patients (Fig 1). Almost half of all patients with a depression had also DRD (46.5%, 318/684).

Tables 1 and 2 show that the prevalence of DRD differed across the health clinics, ethnicity, religion, religiosity, also across having a blood pressure ≤ 130/80 mmHg or dyslipidaemia; whereas depression differed across age, employment, triglyceride level and number of anti-hypertensive agents. The combination of DRD and depression differed across health clinics (χ^2^ = 37.44, *p* < 0.0001), ethnicity (χ^2^ = 13.23, *p* = 0.001), religions (χ^2^ = 19.98, *p* = 0.001), religiosity (χ^2^ = 9.61, *p* = 0.008) and employment (χ^2^ = 6.81, *p* = 0.033).

### Variables associated with DRD

DDS scores correlated moderately with the PHQ scores, spearman’s *r* = 0.50 (*p* < 0.0001); while both DDS and PHQ had a weak negative correlation with age, *r* = -0.16 (*p* < 0.0001) and *r* = -0.09 (*p* = 0.02), respectively; duration of diabetes, hypertension and dyslipidaemia were not correlated with either DRD or PHQ scores.

The final multivariable model showed an association between patients with higher DDS score and younger age, Chinese patients, those attending Dengkil health clinic and those who had higher scores on the PHQ (Table 3). No associations were noted between DRD and HbA1c, blood pressure or lipids.

**Table 3:** Factors associated with diabetes-related distress and depression.

Parameter	Odd Ratio	95% Confidence Interval		*P* value
		Lower	Upper	
Diabetes-related distress, n = 474*				
Age	0.995	0.996	0.991	0.043
Ethnicity (Malay**)				
Indian	1.04	0.958	1.129	0.347
Chinese	1.16	1.040	1.287	0.007
Health clinic (Seri Kembangan HC**)				
Salak HC	0.94	0.843	1.055	0.304
Dengkil HC	1.11	1.003	1.223	0.044
Total PHQ score	1.05	1.040	1.056	< 0.0001
**Depression, n = 416**^†^				
Health clinic (Seri Kembangan HC**)				
Salak HC	0.90	0.726	1.123	0.360
Dengkil HC	0.74	0.598	0.903	0.003
Marital status (Single**)				
Married/living with partner	1.15	0.828	1.596	0.407
Widowed	1.21	0.815	1.789	0.346
Divorced/separated	1.66	1.012	2.731	0.045
Microvascular complications (No**)				
Microvascular complications (Yes)	1.36	1.064	1.732	0.014
Total DDS score	1.03	1.020	1.029	< 0.0001
**Diabetes-related distress and depression combined, n = 488**^‡^				
Health clinic (Seri Kembangan HC**)				
Salak HC	0.28	0.123	0.626	0.002
Dengkil HC	0.97	0.513	1.846	0.934
Employment status (Employed**)				
Unemployed	4.65	1.020	21.204	0.047
Retired/house wife	0.83	0.443	1.540	0.548
BP (BP ≤ 130/80 mmHg**)				
BP > 130/80 mmHg	0.53	0.317	0.891	0.016

AHA = anti-hypertensive agent; BMI = body mass index; BP = blood pressure; HC = health clinic; LDL-C = low-density lipoprotein cholesterol; PHQ = Patient Health Questionnaire 9 items

*Other variables in the model: gender, religiosity, marital status, employment status, income, exercise, BMI, LDL-C, hypertension, dyslipidaemia, blood pressure, anti-hypertensive agent

^†^Other variables in the model: age, gender, ethnic, employment status, exercise, LDL-C, dyslipidaemia, oral hypoglycaemic agents, anti-hypertensive agent

^‡^Other variables in the model: age, gender, ethnic, religiosity, BMI, hypertension, dyslipidaemia, LDL-C, anti-hypertensive agent

**Reference group

For the EB subscale, younger age (OR 0.994, *p* < 0.0001), being Chinese (OR 1.16, *p* = 0.003), higher PHQ scores (OR 1.06, *p* < 0.0001) and receiving medical care at Dengkil (OR 1.17, *p* = 0.002) or Salak (OR 1.11, *p* = 0.045) health clinics were independently related to higher emotional burden.

For the PD subscale, Chinese (OR 1.13, *p* = 0.049) patients, those who were not taking anti-hypertensive agents (OR 1.20, *p* = 0.022), those with higher PHQ scores (OR 1.04, *p* < 0.0001) and receiving medical care at Dengkil (OR 1.41, *p* < 0.0001) or Seri Kembangan (OR 1.28, *p* < 0.0001) health clinics had more physician-related distress.

For the RD subscale, Chinese (OR 1.17, *p* = 0.002) patients, patients with higher PHQ scores (OR 1.05, *p* < 0.0001) and those receiving medical care at Dengkil (OR 1.11, *p* = 0.051) health clinics had more regimen-related distress.

For the ID subscale, we noticed the same determinants in the above model and in a model further adjusted for marital status and education level, patients who were taking one to three anti-hypertensive agents (OR 1.20–1.17, *p* = 0.014–0.038), those with higher PHQ scores (OR 1.05, *p* < 0.0001) and those receiving medical care at Dengkil (OR 1.19, *p* = 0.022) or Seri Kembangan (OR 1.17, *p* = 0.022) health clinics had higher interpersonal distress.

### Variables associated with depression

The final multivariable model showed an association between a higher PHQ score and microvascular complications and between a higher PHQ score and a higher DDS score (Table 3). Compared to those visited Seri Kembangan health clinic, those attending Dengkil health clinic had a lower chance to be depressed (Table 3). Compared to being single, being divorced or separated was associated with depression (Table 3). Depression was not associated with HbA1c, blood pressure or lipids.

### Variables associated with DRD-depression combined

In the model with both distress and depression (Table 3), patients who were unemployed, under medical care at the Dengkil or Seri Kembangan health clinics and those who achieved a blood pressure target ≤ 130/80 mmHg were more likely to experience both distress and depression. With HbA1c and lipids, no association was found.

## Discussion

This study highlights the prevalence and distribution of DRD and several degrees of depression in a representative sample of adult T2DM patients in Malaysia treated at the primary care level [45]. The Malaysian National Diabetes Registry (NDR) 2009–2012 [45] consists of a random sample from 644 public health clinics nationwide. It shows that the mean age of T2DM patients in the NDR was 59.7 years old (compared to 56.9 in this study) and 58.4% were women (52.9 in this study). Ethnicity composition in the NDR showed that 58.9% were Malay, 21.4% were Chinese and 15.3% were Indian compared to this study of 52.8%, 23.4% and 23.7%, respectively. The median of diabetes duration was five years in the NDR compared to four in this study.

DRD was present in a significant proportion of patients and the proportion of DRD seemed to increase in the higher categories of depression. A similar observation was reported in a Polish study [46]. DRD prevalence was much lower in study among the native Dutch T2DM patients (primary care 4%, hospital 13%) but similar to the minority of the non-native Dutch patients (55% Turkish, 40% Suriname and 23% other ethnics) [18]. In mainland China, the prevalence of DRD was 64% among the T2DM patients at two public hospitals [35]. The prevalence of DRD or/and depression in this study was almost doubled compared to the diabetes outpatient clinic at the University Medical Center Groningen in The Netherlands [47].

The overall prevalence of any degree of depression was significantly higher than that in the United States (US) (12%) and Europe (14.7%), but comparable to that in the Iraq (51.8%) [48]. Aikens et al. [20] reported that about half of the US T2DM patients had some degree of depression (PHQ total score > 5) which is similar to our study. Smaller Malaysian studies both at the health clinics (using the DASS questionnaire) [15] and the hospital (using the Hospital Anxiety and Depression Scales [HADS]) [49], reported a depression prevalence of 11 to 12%, comparable to the percentage of patients with moderate to severe depression in the present study. These data indicate that depressive symptoms and DRD vary widely in different countries and healthcare settings. It is possible that there are racial differences in depression [15] and DRD but also the different measures used to assess depression and DRD may cause different prevalence [36]. Also noteworthy was the significant presence of mild depression without DRD in this group of patients. It is possible that many of these patients with depressive symptoms only and not DRD suffer from non-diabetes related problems [16]. A Japanese study in T2DM showed that DRD was significantly related to the severity of depressive symptoms, independent of the severity of complications and glycaemic control [50]. Skinner et. al. [51] reported that DRD (Problem Area in Diabetes [PAID]) in adult T2DM across 17 primary care organizations in England and Scotland, were predictive of depressive symptoms at 12 months. A similar longitudinal study would be needed to determine the causal longitudinal relationship between DRD and (mild) depression in Asian T2DM patients.

Not surprisingly, DRD and depression were positively associated. Younger patients, Chinese patients and those receiving diabetes cares from certain health clinics had significant overall and different types of DRD, as measured by the subscales. Also in other studies younger age was associated with higher DRD [30], possibly due to the impaired active life-styles from the diabetes treatment and the demands of self-care. The mismatch between expectation and reality could be the main reason for the DRD among the Chinese patients. The mismatch between expectations and experiences could result from unexpected difficulties with the healthcare system and anti-diabetes treatment from the public health clinics and doctors, respectively. Chinese patients were known to prefer private healthcare services and practitioners [52]. Thus, it might be possible that they felt distressed when comparing their medical management from the public health clinics to their friends’ or relatives’ private treatment, because it was known that public health clinics were generally caring for more patients, were more crowded, had longer waiting times with medical care more often provided by junior doctors and with less ‘innovative’ (and less expensive) blood glucose lowering drugs prescribed. This “neighbourhood culture”: perceived social and physical order, social and cultural environment, and access to services and facilities, was associated with regimen-related distress and physician-related distress [53].

Living alone as a single was a protective factor against depression, compared to being divorced or separated. Living with diabetes and all its demands involves patients’ spouses too [54], and it is known that coping with changed lifestyles could stretch the strained relationship in a negative direction [6]. Unsupportive gestures, remarks or uncaring attitudes from their spouses could cause psychological burden even resulting in feeling depressed [7]. Obviously living alone as a single, divorced or separated did not burden a partner’s daily life. The fact that even patients separated from their spouses reported to be more depressed compared to the single patients could be the result of the negative influence of other significant others such as children or family members [55]. With appropriate and practical information to the relatives of T2DM patients, they could become the social supports to the patients in adhering to the recommended healthy life-styles [56].

The absence of notable effects of other variables such as exercise, smoking, income, educational status and complications might be due to the lack of variability of these characteristics among the participating patients. No gender effects on either DRD or depression were observed in multivariable analyses, in contrast with the findings from other studies [57]. There was no association between DRD or depression and HbA1c in this study which is in contrast to other studies [58, 59]. No effect of diabetes-related complications on DRD was also reported in both primary and secondary care in The Netherlands [18]. Unfortunately, we were not in the position to collect any other somatic complaints or comorbidities. Indeed, diabetes complications were often associated with depression and/or diabetes-related distress in T2DM. Patients with microvascular complications are more likely to experience depression [60] and also a recent Dutch study at community level with people with T2DM observed a significant relationship between DRD (measured by PAID) and microvascular, but not macrovascular complications [61]. Since it was likely that microvascular complications were emerging in the later stages of diabetes and relatively less challenging at the primary care level because those people with complications would have been referred to secondary/tertiary care at the hospitals. Therefore, the influence of diabetes-related complications on DRD and depression in our study population might be limited [62, 63].

When DRD-depression was combined as an outcome, the effects of some variables became more apparent, especially those of the blood pressure and LDL-C targets. The strength of the association of health clinics, employment status and target blood pressure with the combination of DRD and depression increased considerably compared to the multivariable regression analyses on either DRD or depression. Explanation from the patient’s perspective of the increased risk of the combination of DRD and depressed when receiving care at the Dengkil and Seri Kembangan health clinics requires clinic-level and physician-level evaluations [64]. It is intriguing to discover the opposite effects of achieving blood pressure and LDL-C targets on DRD and depression as a combined outcome. Besides the above mentioned explanations, this finding could be the result of experienced side effects of antihypertensive medication. Although the relation between side effects and distress and depression is not well established, it is obvious that the occurrence of side effects may have an impact on psychological well-being. Unfortunately, data on medication side effects in people from this cohort are not available to demonstrate such a relationship. In addition, the blood pressure target could have been achieved or maintained at the expense of mixed feelings with physicians’ decision on the need of anti-hypertensive agents and self-experimentation of alternative and complementary treatments for hypertension [65], or from distressed interpersonal relationships causing some degree of depression in the process of adhering to the prescribed anti-hypertensive agents [66].

Large sample size and high response rate were the strengths of this study. Non-significant relationships between many self-care behaviours with DRD and depression might be due to insensitive single-item measures, or lack of heterogeneity in the study population. It is possible that there were biases in reporting, especially when patients were interviewed or in the presence of other significant others.

## Conclusions

DRD and depression were more common in Asian adult T2DM treated at the primary care level compared to Western T2DM patients. DRD and depression could be present on their own, but if combined with DRD more patients had more severe depressive feeling. DRD and depression on their own and in combination shared few determinants, such as younger age and Chinese ethnicity, and had many distinctive associated socio-demographic factors. Future work is needed to determine the direction of relationship of these psychological disorders and to investigate the relationship between hypertension targets and DRD-depression.
